# A cross-sectional study on health differences between rural and non-rural U.S. counties using the *County Health Rankings*

**DOI:** 10.1186/s12913-015-1053-3

**Published:** 2015-10-01

**Authors:** Timothy J. Anderson, Daniel M. Saman, Martin S. Lipsky, M. Nawal Lutfiyya

**Affiliations:** Essentia Institute of Rural Health, 502 E. 2nd Street, Duluth, MN 55805 USA; National Center for Interprofessional Practice and Education, University of Minnesota-Twin Cities Campus, MMC 501 Mayo, 420 Delaware St SE, Minneapolis, MN 55455 USA; Roseman University of Health Sciences, 10920 S. River Front Parkway, South Jordan, UT 84095 USA

**Keywords:** Rural health, Health outcomes, Rurality, County Health Rankings

## Abstract

**Background:**

By examining 2013 *County Health Rankings and Roadmaps* data from the University of Wisconsin and the Robert Wood Johnson Foundation, this paper seeks to add to the available literature on health variances between United States residents living in rural and non-rural areas. We believe this is the first study to use the *Rankings* data to measure rural and urban health differences across the United States and therefore highlights the national need to address shortfalls in rural healthcare and overall health. The data indicates that U.S. residents living in rural counties are generally in poorer health than their urban counterparts.

**Methods:**

We used 2013 *County Health Rankings* data to evaluate differences across the six domains of interest (mortality, morbidity, health behaviors, clinical care, social and economic factors, and physical environment) for rural and non-rural U.S. counties. This is a cross-sectional study employing chi-square analysis and logit regression.

**Results:**

We found that residents living in rural U.S. counties are more likely to have poorer health outcomes along a variety of measurements that comprise the *County Health Rankings’* indexed domains of health quality. These populations have statistically significantly (p ≤ 0.05) lower scores in such areas as health behavior, morbidity factors, clinical care, and the physical environment. We attribute the differences to a variety of factors including limitations in infrastructure, socioeconomic differences, insurance coverage deficiencies, and higher rates of traffic fatalities and accidents.

**Discussions:**

The largest differences between rural and non-rural counties were in the indexed domains of mortality and clinical care.

**Conclusions:**

Our analysis revealed differences in health outcomes in the *County Health Rankings’* indexed domains between rural and non-rural U.S. counties. We also describe limitations and offer commentary on the need for more uniform measurements in the classification of the terms rural and non-rural. These results can influence practitioners and policy makers in guiding future research and when deciding on funding allocation.

## Background

There are differences in health, access to and the quality of healthcare between rural and urban areas in the United States [1]. Despite the various methods of classifying what constitutes a rural or urban location [2], studies continue to find that differences in health and the health care of rural populations and their urban peers are real [3–6]. Place is an essential variable in determining disparities between different populations [1, 7]. We used the County Health Rankings (CHR), a project created by the University of Wisconsin Population Health Institute and the Robert Wood Johnson Foundation [8] to further examine the importance of place with regard to health outcomes. The CHR model is framed on the interconnectedness between health outcomes and health factors with policies and programs at the local, state, and federal level. Health factors measure the population health of a county and are comprised of weighted data on (1) health behaviors, (2) clinical care, (3) social and economic factors and the (4) physical environment. The following measures make up the health factors that influence the health outcomes of mortality and morbidity. Table 1 further details the CHR model and includes data sources.

**Table 1:** County Health Rankings Model: Measures and Data Sources

	Measure	Data source
Health outcomes		
ᅟMortality 50 %*	Premature death	National center for health statistics
ᅟMorbidity 50 %	Poor or fair health	Behavioral risk factor surveillance system
	Poor physical health days	Behavioral risk factor surveillance system
	Poor mental health days	Behavioral risk factor surveillance system
	Low birth weight	Behavioral risk factor surveillance system
Health factors		
Health behaviors (30 %)		
ᅟTobacco use	Adult smoking	Behavioral risk factor surveillance system
ᅟDiet & exercise	Adult obesity	National center for chronic disease prevention and health promotion (NCCDPHP)
	Physical inactivity	NCCDPHP
ᅟAlcohol & drug use	Excessive drinking	Behavioral risk factor surveillance system
ᅟSexual activity	STI data	National center for HIV/AIDS, viral hepatitis, STD, and TB prevention
	Teen births	National center for health statistics
Clinical care (20 %)		
ᅟAccess to care	Uninsured	Small area health insurance estimates
	Primary care physicians	HRSA area resource file
	Dentists	HRSA area resource file
ᅟQuality of care	Preventable hospital stays	Medicare/dartmouth institute
	Diabetic screening	Medicare/dartmouth institute
	Mammography screening	Medicare/dartmouth institute
Social economic factors (40 %)		
ᅟEducation	High school graduation	Data.gov, supplemented w/ national center for education statistics
	Some college	American community survey
ᅟEmployment	Unemployment	Bureau of labor statistics
ᅟIncome	Children in poverty	Small area income and poverty estimates
ᅟFamily and social support	Inadequate social support	Behavioral risk factor surveillance system
	Children in single parent households	American community survey
ᅟCommunity safety	Violent crime	FBI Uniform crime reporting
	Injury deaths	CDC Wonder
Physical environment (10 %)		
ᅟAir and water quality	Air pollution - particulate matter	CDC Wonder
	Drinking water violations	Safe drinking water information system
ᅟBuilt environment	Limited access to health foods	USDA food environment atlas
	Fast food restaurants	County business patterns
	Access to recreational facilities	County business patterns

Policies and Programs

Federal, state and local programs believed to target health outcomes directly or attribute to health factors which cause outcomes

*Percentages indicate weights based on comparative importance within an outcome/factor and data quality

1. Health behaviors are measured by tobacco use, diet and levels of exercise, sexual activity and the use of alcohol and drugs.
2. Clinical care is measured by access to and quality of care.
3. Social and economic factors are measured by education, employment, income, family and social support, and community safety.
4. Physical environment consists of air and water quality as well as built environment data (access to healthy foods and recreational facilities).

The literature supports evidence of rural and urban differences in many of the health factors measured by CHR. *Health behavior* research indicates that both adolescents and adults in rural areas are more likely to smoke [9, 10]. Moreover, rural children over the age of five are more likely to be obese or overweight [11], as are rural adults [12]. Rural residents consume fewer fruits and vegetables [13], and have greater rates of alcohol addiction and consumption than in urban locations [14]. Levels of physical activity are also higher in urban areas, with physical inactivity being higher in rural locations, particularly in the American South [15].

*Clinical care* research indicates that often there is a shortage in resources available to rural residents. Rural citizens in the United States are less likely to have health insurance as compared to suburban residents [16]. The Centers for Disease Control and Prevention (CDC) reports that rural citizens have fewer medical specialists per 100,000 people, including less pediatricians, obstetricians/gynecologists, and internists. Only the number of general and family physicians increase along the rural gradient [9]. Dental problems such as tooth loss increase along the urban–rural gradient [17], owing in part to a lack of dentists and dental visits [9]. Regarding quality of care, studies have shown that rural residents have a higher proportion of preventable hospital stays for acute and chronic conditions [18, 19].

While a complete analysis of the *economic and social* situation for rural residents would be a separate piece entirely, we do see evidence that while there has been an increase in rural children attending college [20], urban residents are still broadly more likely to have a bachelor’s degree [21]. High school dropout rates may be similar for the two cohorts [22], as deeply rural and urban areas both suffer from systemic poverty [23].

*Physical environment* is the final of the four health factors affecting health outcomes. A review of the literature on this admittedly wide-ranging topic shows advantages and disadvantages depending on the subject under review. Recent evidence shows that rural areas in the United States have lower air pollution and nitrogen dioxide concentrations [24]. However, rural counties unsurprisingly have greater exposure to agriculture-related pollution than urban counties [25]. Asthma morbidity may also be worse for rural compared to urban patients [26]. With regard to the built environment, access to recreational facilities has been found to be associated with BMI levels. [27–29]. There is also evidence to suggest that some rural residents lack access to such facilities [30].

The CHR model’s county *health factors* ultimately explain a county’s *health outcomes* of mortality (length of life) and morbidity (quality of life). The literature on these two topics supports the causal relationship between factors and outcomes. The CHR uses years of potential life lost (YPLL) for the mortality outcome. The mortality rate for rural residents has been higher since at least 1968, although there appears to be some betterment of these numbers recently [31]. CDC data indicates that unintentional injuries contributing to death, such as poisoning, suffocation, and falls are more likely to occur in rural areas. Furthermore, the motor vehicle fatality rate for rural residents is believed to be almost double that of urban residents, attributed in part due to longer prehospital times [32, 33]. Rural residents often have more hazardous occupations, a fact interconnected with social and economic factors, and experience delays in emergency response. Rural accident victims also have access to a lower number of healthcare facilities [9].

The morbidity outcome is based on health related quality of life and birth outcomes. Rural populations are more likely to have type 2 diabetes mellitus [34], and overall self-ratings of individual health decrease in rural areas [35]. The quality of life measure includes mental health. Research indicates that suicide rates, which affect YPLL, for both males and females were higher for those living in rural areas, with one study indicating rural men having twice the suicide rate when controlling for such variation as the divorce rate and ethnicity [36]. Children in rural areas are more likely to have behavioral and mental health problems as compared to their urban counterparts [37]. Access to mental health care can be limited in rural areas due to transportation problems, lack of reporting mental health issues, and a self-reliance that often includes self-care [38, 39]. When rural citizens do receive mental health treatment, the quality of clinical care has been questioned despite data indicating higher levels of need [40, 41].

The objective of this study was to determine whether differences in the 2013 CHR indexed domains of health outcomes and their causal health factors exist between non-rural and rural counties. To our knowledge, this is the first study to examine differences in health outcomes between rural and non-rural counties utilizing CHR data. There are several benefits to using the CHR. As described above, the model incorporates a vast array of data covering numerous aspects related to the health of the U.S. population. Created by a credentialed team of scientists and public health experts, the rankings have been used by other researchers in peer-reviewed articles to study community health, mortality, and the implications on public policy [42, 43].

## Methods

The authors have nothing to report regarding an ethics statement. Research was ruled exempt by the Essentia Health IRB, the IRB of authors TA and DS. The work did not meet the definition of research with human subjects, based on Office of Human Research Protections (OHRP) guidance.

We used publically accessible 2013 CHR county data (*n* = 3053 counties) which grouped counties into quartiles to “de-emphasize the differences between individual county ranks” [44]. Nearly all counties in the United States are ranked in their respective state based on publically available data. Data used in these rankings include health outcomes, which are a measure of premature mortality (deaths under age 75) and morbidity (overall, physical and mental health), both of which are predicted by health factors. CHR data from 2013 encompasses all counties with data available in the U.S. which are indexed into performance quartiles (where the first quartile are the top 25 % of counties within each state, and the fourth quartile are the bottom 25 % of counties within each state) and compared by the two geographic locales of non-rural (*n* = 1088) or rural (*n* = 1965). County ranks are based on computed and weighted composite scores within each state. A calculated weight represents the importance assigned by CHR. The complete methodology associated with calculating the county ranks are available on the CHR website [44].

To determine if the geographic location of a county was rural or non-rural, the county was linked to a database containing the 2003 rural–urban county continuum codes developed by the Economic Research Service of the US Department of Agriculture [45]. We chose this taxonomy as it is commonly used for research [2]. The 2003 continuum codes form a classification scheme whose purpose is to distinguish metropolitan counties by size and non-metropolitan counties by degree of urbanization and proximity to metro areas. This urban–rural continuum scheme has nine categories for classifying US counties, three that are metropolitan and six that are non-metropolitan. In our analyses, the six non-metro categories for counties were coded as rural and the three metro categories of counties were coded as non-rural [46].

Based on the CHR model described in Table 1, we used CHR’s six indexed domains in our analysis (mortality and morbidity outcomes and the health factors of health behaviors, clinical care, social and economic factors, and physical environment). We first compared the six indexed domains between rural and non-rural counties using the chi-square test for significance at alpha level 5 %, where significance was set at p ≤ 0.05. We also used logistic regression analysis where the dependent variable was rural versus non-rural counties and the six domains accounted for the independent variables. A forward regression modelling strategy was used to determine those variables to be included in the final regression model. Adjusted and unadjusted estimated odds ratios are presented along with 95 % confidence intervals. All analyses were performed using SPSS v.21 (SPSS Chicago, Ill).

## Results

Overall, our analyses revealed health differences between rural and non-rural counties in the U.S. Table 2 shows the six indexed domains by quartile for rural and non-rural counties. Significant differences in each index were observed between rural and non-rural counties, with a greater proportion of rural counties in the fourth (worst) quartile. For example, among mortality, 31.5 % of rural counties and only 13.1 % of non-rural counties were in the fourth quartile (p < 0.001). Within the rural locale, there is also a gradient effect from the 1st quartile of mortality (17.0 %) to the 4th quartile of mortality (31.5 %). There is a similar gradient effect across most domains and for non-rural counties as well. Similarly, a significantly greater proportion of rural counties versus non-rural counties in the 4th quartile was observed for the morbidity (29.1 % vs 17.9 %), health behaviors (27.9 % vs 20.0 %), clinical care (32.2 % vs 12.3 %), social and economic factors (30.4 % vs 15.6 %), and physical environment (27.2 % vs 21.3 %) domains.

**Table 2:** Indexed U.S. County Domain Quartiles by Geographic Locale 2013 U.S. County Health Rankings Data

Indexed Domains^a^	Quartile^b^	Geographic Locale		*P* value for Chi Square
		% Rural *n* = 1965	% Non-Rural *n* = 1087	
Mortality	1	17.0	39.9	<.001
	2	24.9	26.3	
	3	26.5	20.7	
	4	31.5	13.1	
Morbidity	1	22.1	30.8	<.001
	2	24.0	26.8	
	3	24.8	24.4	
	4	29.1	17.9	
Health Behaviors	1	19.3	35.8	<.001
	2	24.3	26.3	
	3	28.4	17.8	
	4	27.9	20.0	
Clinical care	1	14.8	43.9	<.001
	2	24.3	26.2	
	3	28.7	17.5	
	4	32.2	12.3	
Social and economic factors	1	18.1	38.0	<.001
	2	24.3	26.2	
	3	27.2	20.1	
	4	30.4	15.6	
Physical environment	1	25.2	25.2	=.002
	2	23.7	27.3	
	3	23.9	26.1	
	4	27.2	21.3	

^a^Each index is comprised of multiple variables that are given different weights

^b^The first quartile is considered the best and the fourth the worst

Source: Author

Table 3 presents the results of a logistic regression model among rural counties for the indexed domains. For mortality, rural counties were at a significantly increased (estimated OR = 3.110, 95 % CI 2.306, 4.195) odds of being in the worst quartile than the best. Rural counties were also at a significantly increased odds of being in the worst versus the best quartile for clinical care (estimated OR = 5.192, 95 % CI 4.001, 6.738) and social and economic factors (estimated OR = 1.792, 95 % CI 1.328, 2.419). However, rural counties were at significantly decreased odds of being in the worst versus the best quartile for morbidity (estimated OR = 0.712 95 % CI 0.531, 0.955) and physical environment (estimated OR = 0.706 95 % CI 0.555, 0.899).

**Table 3:** Logistic Regression Model for Rural Geographic Locale of U.S. Counties by Indexed Domain Quartile 2013 U.S. County Health Rankings Data

Indexed Domain	Quartile	Adjusted estimated odds ratio (95 % CI)	Unadjusted estimated odds ratio (95 % CI)
Mortality	1	--^a^	--^a^
	2	1.691(1.337, 2.140)	2.220 (1.810, 2.722)
	3	1.955 (1.511, 2.530)	3.000 (2.428, 3.707)
	4	3.110 (2.306, 4.195)	5.647 (4.480, 7.118)
Morbidity	1	--*	--*
	2	.819 (.646, 1.037) ^b^	1.245 (1.015, 1.527)
	3	.646 (.498, .838)	1.421 (1.156, 1.748)
	4	.712 (.531, .955)	2.264 (1.824, 2.811)
Health behaviors	1	--^a^	--^a^
	2	1.028 (.811, 1.302) ^b^	1.707 (1.393, 2.093)
	3	1.278 (.981, 1.666) ^b^	2.950 (2.377, 3.661)
	4	.860 (.649, 1.140) ^b^	2.578 (2.087, 3.184)
Clinical care	1	--^a^	--^a^
	2	2.354 (1.891, 2.931)	2.755 (2.240, 3.388)
	3	3.642 (2.868, 4.626)	4.876 (3.907, 6.063)
	4	5.192 (4.001, 6.738)	7.760 (6.125, 9.831)
Social and economic factors	1	--^a^	--^a^
	2	1.319 (1.042, 1.670)	1.946 (1.587, 2.386)
	3	1.556 (1.192, 2.030)	2.829 (2.289, 3.496)
	4	1.792 (1.328, 2.419)	4.074 (3.264, 5.085)
Physical environment	1	--^a^	--^a^
	2	.741 (.589, .932)	.869 (.706, 1.069) ^b^
	3	.674 (.534, .851)	.914 (.742, 1.126) ^b^
	4	.706 (.555, .899)	1.276 (1.031, 1.580)

^a^Reference category

^b^Estimated Odds Ratio not statistically significant

(Dependent variable is rural/non-rural classification of county)

## Discussion

Our results demonstrate generally poorer mortality, clinical care, and social and economic outcomes for rural versus non-rural counties. Overall, rural counties were more likely to be in the fourth quartile of their respective states than non-rural counties. However, we also found that rural counties compared favorably to non-rural counties for the physical environment and morbidity domains. The largest differences between rural and non-rural counties were in the indexed domains mortality and clinical care. Rural counties had three times the odds of being in the 4th quartile of mortality than non-rural counties, and about five times the odds of being in the 4th quartile of clinical care. In addition, the morbidity domain is comprised in part by mental health data, specifically the number of poor mental health days reported by a survey respondent. Given the research on mental health in rural populations, rural performance in this quartile may be surprising. It is possible that higher scores in the morbidity domain are attributable to other measurements outside of mental health. The difference in mortality found in rural counties may be attributable to a host of causes. One of the domains we believe may be a factor in driving the large difference in mortality is poorer access and worse quality of clinical care in rural counties. There is evidence to suggest that rural residents may also tend to delay the receiving of care, increasing the risk of a poorer health outcome [47] and have fewer providers of care [48].

A lack of healthy eating habits may also be attributable to increases in mortality in rural areas. This could possibly be due to a lack of healthier, low cost eating options [49]. In part because of travel restrictions and cost, rural residents may be more likely to buy food at convenience stores rather than at conventional stores such as supermarkets. “Food deserts,” areas of the country where residents have less access to affordable and healthful food tend to be in rural areas although they can also be present in highly urban populations [50, 51].

Rural counties were also at greater odds of being in the fourth quartile of the social and economic factor domain. This domain in part uses motor vehicle injury data and research has found that rural residents are less likely to survive motor vehicle accidents, in part due to access restrictions [52, 53]. The domain also takes into account education, employment and income factors, where it was found that residents in rural counties were at the lower end on these measurements.

### Limitations

On a conceptual level, there is a lack of agreement between invested parties on what ‘rural’ means and how the term should be defined and measured. This creates problems for policymakers and the health-care providing community [2]. Standardizing the definition and measurement of rurality is a difficult task and likely impossible given the variety of interests on how the terms should be used. The U.S. federal government has multiple definitions for the term [2]. Scholars should choose definitions in line with their research question and available data and resources.

Another limitation is that the CHR does not take into account all possible factors that determine community health. For example, the physical environment domain encompasses multiple factors (air pollution, water quality, the built environment), but it of course does not and cannot account for all possible components that could make up one’s definition of the physical environment. The conceptual framework of the health factors and outcomes are certainly open to critique, and therefore some caution must be used when making statements about study results. However, given the problem of limitations caused by infrequent data reporting or unavailability for certain regions, we believe the CHR team has compiled a respectable dataset. Ultimately, CHR was able to compile data from more than 97 % of all counties in the US.

As this paper is a cross-sectional study, causality cannot be confirmed. Furthermore, the methodology for this paper divided counties into four quartiles based on each state’s quartile rankings. This means that counties in one state may have overall poorer health than counties in other states that have the same quartile rankings. There may be an argument against using quartiles and instead using natural breaks in the entire US county dataset where quartiles are set a-priori, and only counties meeting a predetermined threshold would fall in each quartile making nationwide studies possible. While perhaps problematic in certain respects, the data limitations do not prevent the production of meaningful observations and results.

## Conclusion

Ultimately, our results indicate that there are significant differences in the overall health and health outcomes of rural populations as compared to urban populations. Populations in rural counties tended to score below their non-rural peers in the six indexed domains of health measured by the CHR, although the results of our logit regression indicate better performance for rural counties in physical environment and morbidity scores. This research furthers the evidence of the divide in health and healthcare between the observed rural and non-rural populations. We believe public health professionals and policy makers in the US must continue to consider these differences when implementing programs addressing the needs of a geographically diverse population.
